# Load-Adaptive Practical Multi-Channel Communications in Wireless Sensor Networks

**DOI:** 10.3390/s100908761

**Published:** 2010-09-21

**Authors:** Md. Shariful Islam, Muhammad Mahbub Alam, Choong Seon Hong, Sungwon Lee

**Affiliations:** Department of Computer Engineering, Kyung Hee University, 1 Seocheon, Giheung, Yongin, Gyeonggi 449-701, Korea; E-Mails: sharif@networking.khu.ac.kr (M.S.I.); mahbub@networking.khu.ac.kr (M.M.A.); drsungwon@khu.ac.kr (S.L.)

**Keywords:** wirelss sensor networks, multi-channel, load detection, channel allocation and deallocation

## Abstract

In recent years, a significant number of sensor node prototypes have been designed that provide communications in multiple channels. This multi-channel feature can be effectively exploited to increase the overall capacity and performance of wireless sensor networks (WSNs). In this paper, we present a multi-channel communications system for WSNs that is referred to as load-adaptive practical multi-channel communications (LPMC). LPMC estimates the active load of a channel at the sink since it has a more comprehensive view of the network behavior, and dynamically adds or removes channels based on the estimated load. LPMC updates the routing path to balance the loads of the channels. The nodes in a path use the same channel; therefore, they do not need to switch channels to receive or forward packets. LPMC has been evaluated through extensive simulations, and the results demonstrate that it can effectively increase the delivery ratio, network throughput, and channel utilization, and that it can decrease the end-to-end delay and energy consumption.

## 1. Introduction

A wireless sensor network consists of battery-powered sensing devices that transmit their observations to the base station. The sensing nodes have a limited transmission range, so nodes away from the base station deliver their data through intermediate nodes. The data generation rates of the sensing nodes depend on the applications. An elastic application might use varying data rates. For example, a monitoring application generates data at a very low rate in the absence of an event, whereas a particular feature might lead to a huge traffic burst [1]. Because of the limited capacity of nodes, the generated data often exceed the network capacity, leading to congestion and contention loss. A congestion control mechanism alleviates the congestion by restricting the nodes from generating data that the network cannot deliver. This ensures optimum usage of the resources and decreases congestion losses. However, if the application requires higher data rates, a congestion control (or rate control) mechanism cannot meet the demand. Therefore, some works have advocated for increased network resources to avoid congestion and to deliver the required data [2,3].

On the contrary, recent sensor motes, such as MicaZ [4] and Telos [5], are capable of using a number of channels [6]. A single adapter can use different channels at different times. If nearby nodes use orthogonal channels, multiple nodes can transmit simultaneously, thereby increasing the network capacity. Multi-channel communications can provide the required data delivery without adding extra resources. The use of a single channel is, therefore, not only an under-utilization of the limited resources of WSNs, but it might also hinder the fidelity of the application.

To improve the network capacity, many multi-channel medium access control (MAC) protocols have been proposed. These protocols generally assign (as part of the network setup) orthogonal channels to the nodes (either to the senders or the receivers) in a two-hop neighborhood [7–9]. The data transmissions among neighbors, therefore, require channel switching and a sophisticated MAC scheme to find a rendezvous time for the sender and receiver. As a result, such protocols require fine-grained time synchronization among the nodes.

To minimize the channel switching and to use multiple channels when necessary, a recent paper proposes a dynamic channel allocation policy based on control theory (hereafter referred to as DM-MAC) [10]. Because the nodes in DM-MAC change channels in a distributed manner for multihop communications, the nodes still need to switch channels. In order to completely avoid channel switching, a static channel allocation policy is proposed in TMCP [11]. TMCP divides the network into a number of sink-rooted sub-trees, where each sub-tree uses an orthogonal channel. However, the sub-tree creation requires a costly initialization phase. However, the sub-tree creation requires a costly initialization phase.

In this paper, we design a multi-channel communications system for WSNs. LPMC dynamically adds or removes channels based on the active network load, and uses multiple channels whenever (when the network load is higher than the capacity) and wherever (the part of the network with a high load) it is necessary. While LPMC has a similar flavor in terms of channel switching to DM-MAC [10], and in terms of channel allocation to TMCP [11], it differs considerably in the following ways:

- Unlike TMCP, LPMC does not need any initialization, such that the overhead is reduced. LPMC assigns channels dynamically instead of the static allocation of TMCP. TMCP divides the network into sub-trees by considering the equal data rate of the nodes. Due to the dynamic channel allocation, LPMC is transparent to data rates.
- Unlike DM-MAC, LPMC adds or removes channels based on the overall network load. Furthermore, the sink controls the channel changing instead of the sensor nodes, since it has a more comprehensive view of the overall network traffic.

The main contributions of this paper can be summarized as follows: (i) We propose a multi-channel communication systems for WSNs that keeps the protocol functionalities out of the sensor nodes as much as possible. (ii) LPMC dynamically identifies the network load and adds channel(s) to the mostly heavily loaded part. No initialization steps are required for LPMC, and the overhead for channel assignment is minimal. Nodes do not need to switch channels to receive or forward packets. (iii) LPMC dynamically adds paths with non-interfering channels to a set of nodes to meet the traffic demands. (iv) The performance of LPMC is evaluated through extensive simulations, and the results demonstrate that LPMC performs better than the existing schemes in terms of packet delivery ratio, network throughput, end-to-end delays, and energy consumption.

The rest of the paper is organized as follows. In Section 2, we explain the existing multi-channel mechanisms for WSNs. We present the proposed mechanism in detail in Section 3. Section 4 demonstrates the performance evaluation of the LPMC. Finally, we conclude in Section 5 with a direction to the future works.

## 2. Related Works

In the existing literature, a significant number of MAC protocols (such as [12–15]) have been proposed for multi-channel communications in wireless networks. However, most of these protocols are not suitable for WSNs, because they assume that the transceiver can operate on multiple frequencies simultaneously or that the nodes are equipped with multiple radios, and current sensor nodes with only a single half-duplex radio transceiver cannot satisfy those assumptions.

The idea of multi-channel protocols in WSNs is not new. A number of MAC protocols have already been proposed for WSNs [7–9]. To achieve multi-channel diversity, most of these protocols assign different channels to the contending sender-receiver pairs. The receivers (or the senders) in a two-hop neighborhood are assigned different channels in order to avoid interference and to increase capacity. However, due to multi-hop communications in WSNs, the nodes need to receive and forward packets in different channels. Therefore, the nodes frequently switch channels and experience packet losses. The channel switching causes considerable delays and a high degree of synchronization. Furthermore, the nodes require a sophisticated scheduling mechanism in order to find the rendezvous time for the sender-receiver pair.

A dynamic channel allocation method is proposed in DM-MAC [10] that uses a control theory approach to dynamically allocate the channels to each sensor. Initially all the nodes communicate on the same channel and when a channel becomes overloaded, nodes migrate to new channels. More specifically, whenever a channel becomes overloaded, some of the nodes switch to other non-interfering channels. Nodes in DM-MAC measure the success rates of medium access. Once a node figures out that lot of messages are lost due to collisions and interference, and causes the success rate of the current channel to fall below a certain threshold, the node considers switching channels. In contrast, nodes return to the previous channel if the success rate increases. Therefore, in a lightly loaded condition, the nodes use a single channel. In high network load conditions, the nodes use multiple channels to increase the network capacity and to deliver the data. However, the main problem with that mechanism is that the nodes change channels independently. In multihop communications, the forwarding nodes might find that the next hop is using a different channel. Therefore, nodes in a single path might need to switch between channels. Channel switching causes delays, and the overall throughput might suffer. In addition, the neighboring nodes in a path require time synchronization, and it is a challenge to keep both the sender and receiver in the same channel.

To avoid channel switching, TMCP divides the network into a number of sink-rooted disjoint subtrees [11]. Nodes residing on different trees are assigned different channels. Each sub-tree uses an orthogonal channel, and, thus, the nodes do not require channel switching. Note that instead of assigning channels to the nodes like DM-MAC, TMCP assigns channel to the sub-trees which allows TMCP to work with a small number of channels. The goal of TMCP is to partition the network to experience minimum intra- and inter-tree interference. The inter-tree interference is eliminated by using orthogonal channels for different sub-trees. In contrast, network partitioning minimizes the intra-tree interference. Finally, a greedy heuristic is used to partition the network to replace the NP-complete partitioning problem.

However, TMCP has a heavy initialization phase that is required in order to partition the network. The tree partition does not consider the data rates of the nodes, so the sub-trees might have different loads. Furthermore, changes in the routes might require a reinitialization, which is too costly for WSNs. Moreover, if a set of nodes sends data at a very high rate, static channel allocation cannot deliver the data. Therefore, we propose a dynamic channel allocation method, in which the forwarding nodes do not require channel switching and channels are added or removed wherever and whenever necessary.

## 3. Proposed Mechanism

### 3.1. LPMC Overview

To describe LPMC, we introduce the following notation and terminology. We define the base station (*i.e.*, sink) as an entity that collects data from the sensor nodes (sources). We consider that the sensor network is mainly used for data collection. The data collection scheme builds a tree that connects the sink and the nodes. Each node forwards the data along the tree.

It is obvious that the use of multiple channels increases the network capacity. The base station needs to sink (receive) all of the data sent in different channels. We, therefore, assume that the sink is equipped with multiple transceivers, each of which works in a different channel. A single node can generate data for multiple concurrent applications. The data of a particular node uses a single path. We assume that there are *K* orthogonal channels available for the WSN. A detailed discussion on the number of effective channels that can be used in WSNs with the CC2420 radio chip can be found in [11].

Our proposed mechanism, LPMC, aims at utilizing minimum number of channels and channels are dynamically added if particular channels become overloaded. If a single channel is sufficient, then it uses one channel. If the generated data requires more capacity, channels are added. In contrast, when the traffic does not need additional channels, channels are removed so that the nodes eventually use only one channel. Whenever a channel is added, it is assigned to a set of nodes in the overloaded tree branch and thereafter, data flows in an entire path uses a single channel. Therefore, nodes in a single path do not need to switch channel to receive data from the upstream nodes, or to forward data to the downstream node.

An important design consideration for LPMC is to allocate channels based on the network load. Therefore, we need to effectively estimate the active load of the network and to allocate or deallocate channels accordingly. In generic WSNs, the sensor nodes only have a local view of the overall network behavior. They might effectively measure the traffic load in a neighborhood, but they are not well-positioned to perceive the overall network status [16,17]. In contrast, a sink has a more comprehensive view of the overall network performance, since it receives the data generated by all of the sources. Given this perspective, a sink can operate the channel management functionalities more efficiently than would be possible with a decentralized approach. We therefore keep the *channel management functionality* of LPMC at the sink, whereas sensor nodes are engaged only in changing the channels. The term *channel switching* refers to the interchange between channels by a node to receive and forward packets, whereas *channel changing* refers to the assignment of a new channel to a set of nodes.

At the sink, LPMC has four distinct logical components:

- The *network load detection* (NLD) component observes the packet arrival rates and sending rates of the sources, and decides whether or not the network is overloaded.
- The *channel allocation and deallocation* (CAD) component adds or removes channels based on NLD’s report about the network load.
- The *path update* (PU) component dynamically divides a group of nodes using one channel into two groups, and assigns a new channel to one of the groups if a single channel is unable to deliver the data of all of the nodes.
- The *channel changing* (CC) component sends an explicit message to the nodes to change linebreak their channel.

The design of LPMC does not depend on any features specific to a particular MAC layer, except for changing the operating channel. Link-level retransmission can improve the performance of LPMC, but it is not essential. We assume that the sensor nodes run a routing protocol that selects a path from each source to the sink. In the following sections, we describe the detailed design of LPMC.

### 3.2. Multi-channel Communications System

**Figure 1.**
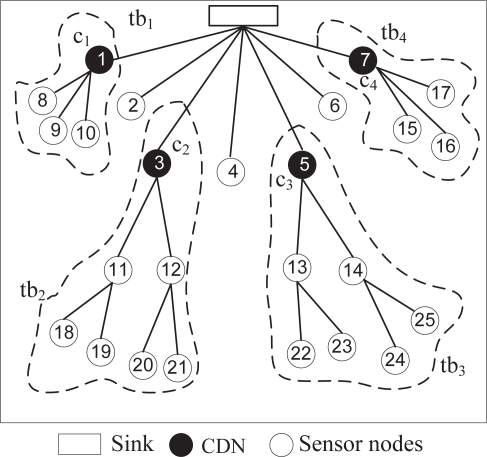
A typical WSN scenario with 4 TBs rooted at 4 CDNs.

The basic idea of LPMC is that all nodes in a single path use the same channel. Nodes do not need to switch channels in order to receive and forward packets. Therefore, in a tree-structure, the channel used by a neighbor of the sink (one-hop away node) is to be used by all of the nodes that forward their data through this node. We refer to this node as the *channel deciding node* (CDN). Figure 1 shows a typical WSN environment, in which a set of nodes sends data to the sink using a tree. There are four CDN nodes in the figure (*c*_1_ − *c*_4_). The nodes that forward their data through a CDN create a *tree-branch* (TB), and all nodes within a TB use the same channel. When a channel assignment takes place, all of the nodes in a TB change channels. However, multiple TBs can use one channel. Figure 1 shows four TBs (*tb*_1_ − *tb*_4_) rooted at four CDNs.

When the network starts, all of the nodes use a predefined channel. We refer to this channel as the *primary channel*. If the primary channel is overloaded, the most heavily loaded TB of the network is assigned a new channel. This continues as long as a channel is available and one of the allocated channels is overloaded. If there is no available channel, we assume that a rate control mechanism will restrict the data rates of the sources in order to avoid the packet losses. The rate control mechanism is beyond the scope of this paper. However, interested readers can refer to [16] and [18], where two well described rate control mechanisms for WSNs can be found. In contrast, when the network load decreases, the added channels are removed. If a single channel can handle the loads of two or more channels, the lowest channel ID is assigned to all of the nodes using these channels.

### 3.3. Network Load Detection (NLD)

One important technical challenge for LPMC is the design of a mechanism to estimate the active load or congestion level in the network or in a TB. Many techniques in the literature of wireless networks or wireless sensor networks measure the congestion level or load at a node. These techniques either measure the channel utilization around a node [19], the forwarding and reception ratio of a node [20], the buffer occupancy at the node [21,22], or a combination of both [18]. In contrast, LPMC estimates the network load from a different point of view. It assumes that the network is not loaded as long as the application’s *reliability* is met. We define the reliability of a WSN application as the ratio of the number of packets received by the sink to the number of packets sent by the sources. Furthermore, LPMC aims at estimating the active load at the sink.

LPMC’s load detection mechanism is based on the following intuition: a network (or a part of it) is not heavily loaded as long as the packet loss rate is acceptable, which permits packet losses due to a poor wireless link, medium contention, and transient congestion. When the network load increases, the packet loss rate also increases, or the interval between successive losses decreases. LPMC, therefore, uses the *average loss interval* as an active network load indicator.

The sink maintains a list of flows for each TB, and it maintains a per-flow list of missing packets and received packets based on the sequence number of the packets. The packets of a flow are forwarded in a single path, so the reception of an out-of-order packet indicates a packet loss. The sink also measures the number of successfully received packets before a loss event in order to measure the average loss interval. LPMC keeps track of the last *n* losses. Suppose, the sequence number of the packets of the *m*-th and the (*m* + 1)-th loss events of the *i*-th flow are *s_m_* and *s_m_*_+1_, respectively. Denoting *d_i,m_* as the length of the *m*-th loss interval of the *i*-th flow, we have *d_i,m_* = *s_m_*_+1_ − *s_m_*.

There are many techniques in the literature for measuring the average loss intervals. However, we choose the *weighted average loss interval (WALI)* method discussed in [23] over the others, because of its robustness in the parameters choices [16]. Therefore, for the last *n* losses, the average loss interval for flow *i*, denoted by *d̂_i_*, is calculated as

(1) $$\begin{array}{c} {\hat{d}}_{i}(1,n)\;=\;\frac{\sum {}_{m=1}^{n}d_{i,m}w_{m}}{\sum {}_{m=1}^{n}w_{m}},\\ \hat{d_{i}}(0,n-1)\;=\;\frac{\sum {}_{m=0}^{n-1}d_{i,m}w_{m}}{\sum {}_{m=1}^{n}w_{m}},\\ \hat{d_{i}}\;=\;\text{max}\;\left[\hat{d_{i}}(1,n),\hat{d_{i}}(0,n-1)\right], \end{array}$$

where *d_i_*_,0_ is the number of successfully received packets since the most recent loss, and *w_m_* is the weight assigned to the *m*-th loss interval. We have used *n* = 10, and *w_m_* = 1/*m* as our parameters. Furthermore, we assume that a smaller value of *m* indicates a recent loss interval, such that the parameters give greater weight to the recent loss intervals than to distant loss intervals.

The reliability of the *i*-th flow, denoted as *r_i_*, can be calculated as *r_i_* = 1 − 1/*d̂_i_*. If *r_i_* is less than the required reliability of the application, *R_req_*, for any flow, then we say that the channel used by the *i*-th flow is overloaded.

However, if the application is loss intolerant (e.g., structural health monitoring application [24]) and lost packets are recovered by end-to-end retransmissions, then the required reliability is 1.0, and we cannot compare it with *r_i_*. Therefore, we assume that the network is not overloaded as long as the loss rate is below a certain threshold. Furthermore, if a lost packet is recovered within the next *n* loss intervals, then we assume that the packet is not lost. Therefore, the sink maintains the history of the last *n* + *q* losses. If the *m′*-th lost packet is recovered by end-to-end loss recovery where 1 ≤ *m′* ≤ *n*, the loss intervals of the last *n* losses are changed in the following way:

(2) $$d_{i,m}\;=\;\{\begin{array}{ll} d_{i,m}, & m\;<\;m\prime \\ d_{i,m}\;+\;d_{i,m+1}, & m\;=\;m\prime \\ d_{i,m+1}, & m\prime \;<\;m\;\leq \;n\;+\;1. \end{array}$$

If the loss rate of any flow exceeds the threshold, then we say that the channel used by the flow is overloaded.

The NLD also measures the active load of the network (*i.e.*, the number of packets sent per unit time). The sink uses a timer for this. When the timer expires, it finds the sequence number of the most recently received packet of each flow and restarts the timer. If the two most recently recorded sequence numbers of the *i*-th flow are *s*_1_ and *s*_2_, then the number of packets sent for the flow is *S_i_* = *s*_2_ − *s*_1_. The current load of a tree-branch, *curr_load*[*tb*], consisting of *F* flows is: $\sum {}_{i=1}^{F}\;S_{i}$. The average instantaneous load, *avg*_*load*[*tb*], of a TB is measured by using the exponentially weighted moving average (EWMA) method as shown:

(3) avg_load[tb] = α × curr_load[tb] + (1 − α) × avg_load[tb],

where *α* is a tuning parameter that is used to smooth the value of the average load of a TB. Through extensive simulation, we have set the value of *α* to 0.12 which produces the best estimation for a long-term average TB load.

### 3.4. Channel Allocation and Deallocation (CAD)

The channel allocation and deallocation component assigns the channels for the TBs. When an overloaded channel is used by multiple TBs, CAD assigns a lightly loaded or unused channel to one of the TBs. LPMC aims to use the minimum number of channels needed to satisfy the traffic load, so it first finds a lightly loaded channel that can be allocated to the overloaded TB (*i.e.*, the TB with the highest loss rate). If it does not find such a channel, then an unused channel is allocated for the overloaded TB. However, if an overloaded channel is used by only one TB, then it cannot allocate another channel to the same TB, as in LPMC, all of the nodes in a TB should use the same channel. In this case, the *path update* component adds a new path by dividing an overloaded TB into two TBs, and assigns a new channel to one of the TBs.

In contrast, if the network becomes lightly loaded after having been at an overloaded status, then it removes one or more of the added channels. The idea of removing a channel is that the unused channel can be allocated to other nodes if necessary. A static channel allocation mechanism cannot do so. More specifically, if there is a continuous source of external interference for any channel, then the delivery ratio of the nodes that use this channel will be very low. A dynamic channel allocation mechanism can easily overcome this problem. First, because the sink keeps track of the achievable data rates of individual channels, it can determine if the loss of data packets is due to some reason other than overloading and can reallocate a separate channel. Second, even if the sink cannot compare it with the maximum achievable capacity, it adds a new channel for the nodes, which at least reduces the load of the channel. Therefore, LPMC tries to shrink the number of used channels. If two or more channels are shrink, the allocation mechanism keeps the channel with the smallest id as the active channel. The channels with higher id’s are removed.

The CAD mechanism maintains two lists: the channel list with the fields < *channel_id*, *status*, *max_load*, *curr_load* and *rem_load* >, and the TB list with the fields < *tb_id* and *avg_load* >. The *status* of a channel is either *used* or *unused*. The CAD periodically obtains the average load (*avg_load*) of each TB from the NLD. The current load (*curr_load*) of a channel is the sum of the loads of the TBs using this channel. The maximum load (*max_load*) of a channel is the highest load that has been supported by the channel so far. When a channel is overloaded, the *max_load* is updated by the CAD; if the *curr_load* is higher than the *max_load*, *curr_load* becomes the *max*_*load* of the channel. The remaining load (*rem_load*) of a channel is the difference between the *max_load* and *curr_load*.

**Algorithm 1:** Channel Allocation and Deallocation (CAD)

1:	Input: *status*[ ], *max_load*[ ], *curr_load*[ ],
2:	*rem_load*[ ], *avg_load*[ ]
3:	ChannelAllocation (Channel *i*) {*i*-th channel is overloaded}
4:	Find the no of TBs, *N*, those use channel *i*.
5:	**if***N* ≤ 1 **then** Call path update component and **return**.
6:	Find the TB with maximum loss rate, *tb*.
7:	**for** Each used channel *j* = 1 TO *K*, Except channel *i***do**
8:	**if***avg_load*[*tb*] ≤ (1 − *β*) × *rem_load*[*j*] **then**
9:	Assign channel *j* to *tb* and **return**.
10:	**end for**
11:	**if** unused channel available **then** assign it to *tb*.
12:	ChannelDeallocation ()
13:	**for** Each Channel *i* = 1 TO *K***do**
14:	**for** Each Channel *j* = *i* + 1 to *K***do**
15:	**if***curr_load*[*i*] + *curr_load*[*j*] ≤ (1−*β*) × *max*_*load*[*i*] **then**
16:	Assign Channel *i* to the TBs using Channel *j*
17:	*curr_load*[*i*] += *curr_load*[*j*]
18:	*status*[*j*] = *unused*
19:	**end if**
20:	**end for**
21:	**end for**

Algorithm 1 shows the detailed operation of CAD. The NLD component notifies the CAD about an overloaded channel. The channel allocation mechanism first checks the number of TBs that use the overloaded channel. If a single TB is using the overloaded channel, then CAD calls the path update component (see next sub-section). If there are multiple TBs, then CAD first tries to allocate a *used* channel; otherwise CAD allocates an *unused* channel if there are any. The channel allocation mechanism finds the TB that has the maximum loss rate. CAD tries to find a used channel with a remaining load that can accommodate the average load of the TB with the highest loss rate. However, the remaining load of a used channel is an estimated value, and an imprecise estimation can cause that channel (*i.e.*, the channel which will be assigned) to be overloaded again. This might enforce another channel assignment. To avoid this, we have used a safeguard, *β*, which ensures that a certain percentage of the remaining load of a used channel is not considered when it is allocated. In the simulation, we have set the value of *β* to be 0.1.

In contrast, the channel deallocation mechanism removes channel when the load decreases. The NLD indicates when a channel is overloaded and CAD runs the *ChannelAllocation* function, whereas every time NLD updates the load of the TBs, CAD runs the greedy *ChannelDeallocaton* function. The ChannelDeallocation function checks whether a single channel (in addition to its current load) can accommodate the current load of another channel or not. If it finds such a channel, then, that channel is allocated the load of both the channels and the other channel is marked as unused.

The capacity of a channel might decrease for to many reasons, for example, very bad link quality, external interference, or even jamming by malicious nodes. LPMC changes the channel if the overall capacity of the channel is decreased to a certain fraction of the *max_load* of the channel. In this case, CAD assigns an unused channel for the TB(s). This feature of LPMC has an inherent benefit over static channel allocation schemes (*i.e.*, TMCP) where it is not possible to dynamically measure the channel capacity and switch to an unused channel.

### 3.5. Path Update (PU)

In LPMC, a single TB uses only one channel, which ensures that a node does not need to switch channels to receive or forward packets. Therefore, whenever an overloaded channel is used by only one TB, CAD cannot allocate a new channel to that TB. A single TB using one channel can be overloaded due to many reasons, which include: (i) randomness of the node deployment, which place many nodes in a small area, (ii) dynamic path selection of the routing protocol, and (iii) nodes from a small portion of a large-scale dense network (usually far away from the sink) generating data at a very high rate. In such cases, LPMC partitions an overloaded TB into two TBs, and assigns a new channel to the newly created TB (*i.e.*, a new TB consisting of some nodes of the overloaded TB). Therefore, paths are updated for a group of nodes in the overloaded TB, which will now use a new CDN to reach the sink. The path update component needs to find leaf nodes (*i.e.*, a node that does not forward the data of other nodes) through which paths to the sink can be established. In case of failure to find such a node, we assume that a rate control mechanism is in place to restrict the source rates which will eventually decrease the channel load.

The *path update* module is initiated from the sink when the sink learns that an overloaded channel is used by a single TB. The sink first sends a unicast *path update message* (PUM) to the CDN of the overloaded TB. The PUM contains the following fields: < *source, destination*, *type*, *tb_ID* >. The *source* is the address of the PUM sender/forwarder, and *destination* is the address of an upstream node of the sender. LPMC assumes that every forwarding node keeps a list of its upstream nodes. The forwarding of a PUM is controlled by the value in *type* field. The *type* field has the value 1 or 2, and sink sets the type value to 1 while initiating a PUM. After receiving a PUM, a node either forwards it (when *type* value is 1) to its upstream node(s) or generates a PUM reply (when *type* value is 2). There may be cases where a PUM forwarder has multiple upstream nodes. In such cases, the PUM forwarder changes the *type* value to 2 and forwards the PUM to a randomly selected half of the upstream node(s). Because two or more branches join in this node, LPMC creates a new path for one of the branches and creates a new TB.

If a node receives a PUM with *type* value 2, it is forced to broadcast a *path update message reply (PUMR)* message. This PUMR creates a path from the node (*i.e.*, PUMR generator) to the sink. The PUMR has the following fixed fields set by the source of the PUMR: < *source*, *destination*, and *tb_ID* >. The *source* is the address of PUMR generator, *destination* is the broadcast address, and *tb_ID* is copied from the PUM. Every PUMR forwarding node (including the PUMR generator) appends the following fields to the PUMR: < *forward_node_addr, channel_ID*, and *hop* >, where *forward_node_addr* is the address of the PUMR forwarder, *channel_ID* is its current channel, and *hop* is the hop count of the node from the sink.

Using the PUMR messages, the PUMR generator tries to find leaf nodes that can further forward the PUMR. However, such a node might be using a different channel. Therefore, PUMR is broadcasted in all of the channels one after another in order to find leaf nodes. The only leaf nodes that can forward a PUMR message are those with a hop count to the sink that is not greater than the value of the *hop* field in the last entry of the PUMR. A node broadcasts the PUMR in a channel and hears the channel for some period. If no leaf node forwards the PUMR within this period, it broadcasts in another channel. Therefore, nodes first forward the PUMR in the receiving channel to ensure that the upstream node (previous broadcaster) hears it. Eventually, the PUMR is received by the sink. If the sink receives multiple PUMRs, it chooses the shortest path. The reverse path appended in the PUMR creates a new TB, and CC assigns a new channel.

**Figure 2.**
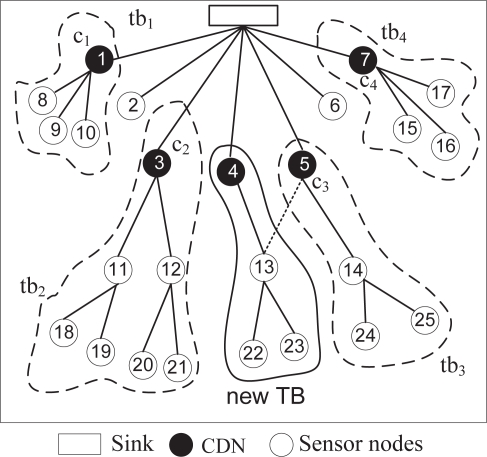
The path update module creates new paths for nodes 13, 22 and 23, by creating a new TB through node 4, and thus, divides the overloaded *tb*_3_ of Figure 1 into two TBs.

We illustrate the path update operation with an example shown in Figure 2. Suppose that a channel is overloaded and is only being used by *tb*_3_. The sink first sends a unicast PUM to node 5, which is the CDN of *tb*_3_. Node 5 has two upstream nodes (*i.e.*, 13 and 14), which means that two branches join in this node. It decides to create a new path for one of the branches. It randomly selects node 13 and sends a PUM with the type value set to 2. Therefore, node 13 becomes the PUMR generator and broadcasts PUMR in different channels to find leaf nodes with a hop count to the sink that is not greater than node 13’s hop count to the sink. Node 4 is such a node, and the PUMR is eventually forwarded by node 4 to the sink. The reverse path appended in the PUMR creates the path from sink to the PUMR generator and creates a new TB. Node 4 becomes the CDN of the new TB, since it is one-hop away from the sink. The new paths from nodes 13, 22 and 23, now go through node 4 to reach the sink. Finally, the sink initiates a control message that enables all of the nodes in the new TB to change their channel. We discuss the channel changing procedure in the next sub-section.

### 3.6. Channel Changing (CC)

The channel changing (CC) component that resides in the sink issues explicit control messages that trigger all nodes in a overloaded TB to change their channel. Two types of *channel changing messages (CCM)* are used: (i) CCM-1 changes the channel of all nodes in a TB, and (ii) CCM-2 assigns a new channel in response to a path update.

To change the channel of an overloaded TB, the sink sends a unicast CCM-1 to the CDN of the TB. The message has the fields < *sender*, *receiver*, and *new_channel* >. All nodes broadcast the CCM-1 except the sink. After receiving a CCM-1, every node checks the *sender* field. If a node receives the CCM-1 from its downstream node, it forwards CCM-1; otherwise, it discards it. While forwarding the CCM-1, each node replaces the *sender* field with its own address and puts the broadcast address in the *receiver* field. When a forwarder hears that at least one of its upstream nodes has forwarded the same message (by snooping), it changes its own channel to the *new_channel*. However, a leaf node changes to the *new_channel* after receiving the CCM-1 from its downstream node.

The CCM-2 message is unicasted with the fields < *sender*, *receiver*, *new_channel*, and *destination* >. The *destination* is the address of the PUMR generator. CCM-2 also includes the reverse path from the sink to the *destination*, and the channel ID of each intermediate node. Every node forward the CCM-2, and changes its channel to the *new_channel*. When the *destination* receives the CCM-2, it converts it to CCM-1 and broadcasts it. Therefore, the path update and channel changing happen simultaneously.

Because the CCM-1 messages are broadcasted, the nodes that are missing this message need to find the channel ID of the downstream node. LPMC uses a *What Is* (WI) message to find the channel ID of a specific node. The node with the ID given in the WI message replies with a *What Is Reply* (WIR) message.

## 4. Performance Evaluations

### 4.1. Simulation Environment

We have performed extensive simulations to evaluate the performance of LPMC in NS-2 [25]. We have considered a network with an area of 200 m × 200 m and 250 nodes placed in a uniform random distribution. We have set the transmission power in such a way that the interference range becomes only 1.5 times the transmission range. In our experiment, the transmission and interference ranges of the nodes are set to 30 m and 45 m, respectively. Actually, this communication model is typically used to simulate the RF model of the CC2420 radio that operates on multiple-channels [11]. The link bandwidth is set to 250 kbps, and 6 orthogonal channels are used. Though the CC2420 radio chip used in Micaz motes provides 16 orthogonal channels, not all of them can be used in parallel because of close channel interference [26]. We use CSMA/CA as the MAC protocol with a maximum of 4 retransmissions. We compare the performance of LPMC with DM-MAC [10] and TMCP [11]. The required reliability (or success rate) is set to 0.95 for DM-MAC and LPMC. However, we set the required reliability of all the mechanisms to 1 when end-to-end reliability is considered. We have performed three different sets of experiments to evaluate the performance of the compared protocols. We show the impact of increasing the offered loads and varying the node densities in the first two set of experiments. Finally, we also show the impacts of the channel quality and external interference. All of the simulations were run 50 times, and the average results are plotted in the graphs. The other system parameters used in the simulation are summarized in Table 1.

### 4.2. Performance Metrics

We have considered the following performance metrics to evaluate the performance of LPMC in a different set of experiments: i. Network throughput- sum of the sizes of the total data packets received by the sink in a unit time, ii. Packet delivery ratio- the ratio of the total number of packets received by the sink to the number of packets sent by the sources, iii. Average end-to-end delay- the average end-to-end forwarding delay (which also includes medium access and switching delay) of the successfully delivered packets, and iv. Average energy consumption- the average energy consumed to successfully deliver a byte of data.

### 4.3. Simulation Results

**Figure 3.**
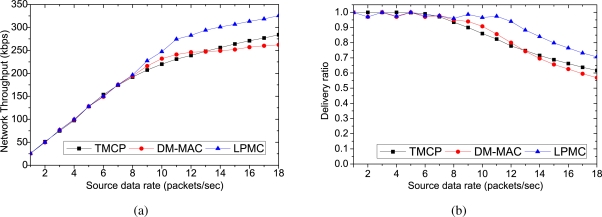
Performance comparison for randomly selected 50 sources with different data rates: (a) network throughput, (b) delivery ratio.

**Figure 4.**
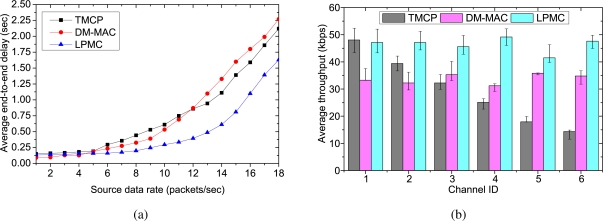
Performance comparison for randomly selected 50 sources with different data rates: (a) average end-to-end delay, (b) average channel capacity when all nodes achieve a fair throughput.

Figures 3 and 4 show the performance comparison for 50 randomly selected sources. We have used the same set of sources for each mechanism. We have gradually increased the source data rates to measure the impact of increasing traffic load on the performance metrics. Figure 3(a) shows the network throughput with increasing data rates. When the source data rates are small (*i.e.*, up to 6 packets/s, all of the mechanisms perform almost equally, because the network remains lightly loaded. However, as the offered load is increased, the channel capacities are exceeded and the performances of the respective protocols start to vary. Due to the static channel allocation, the throughput of TMCP depends on the locations of the sources. The channel with the highest number of nodes is overloaded, while many other channels remain underloaded. The dynamic channel allocation of DM-MAC achieves a higher throughput than TMCP until all of the channels in TMCP are overloaded. LPMC achieves the maximum throughput because it does not require channel switching and it allocates channels dynamically. Furthermore, the fair throughput of the sources (*i.e.*, each source achieves the reliability) in LPMC is achieved up to a source rate of 11 packets/sec where the network throughput is 274 kbps. DM-MAC and TMCP achieve fair throughput up to source rates of 9 packets/s (where the network throughput is 216 kbps) and 7 packets/sec (where the network throughput is 174 kbps), respectively.

Figure 3(b) shows the delivery ratio at the sink, and as expected, LPMC outperforms the other two mechanisms at higher traffic loads. In LPMC, whenever a channel used by a single TB is overloaded, it updates the paths (*i.e.*, it partitions the overloaded TB into two groups and creates a new TB that connects one group to the sink) and assigns a lightly loaded or unused channel to the new TB. Therefore, the number of packet losses due to congestion and contention decreases, and LPMC achieves a higher delivery ratio than TMCP and DM-MAC, while increasing the offered load. At higher traffic loads, DM-MAC experiences more intra-flow interference and therefore, DM-MAC’s delivery ratio becomes less than TMCP’s delivery ratio.

Figure 4(a) shows the average end-to-end delays experienced by the successfully delivered packets. When the traffic load is low, all of the mechanisms experience small delays because the channels are operated with tolerable loads. Moreover, with a small amount of traffic, the channels do not become overloaded, which results in minimal channel switching (if any). However, as the traffic load is increased, LPMC outperforms both DM-MAC and TMCP in terms of the average end-to-end delay. This is because in LPMC, the nodes in a single path do not need to switch their channels to receive or forward a packet. TMCP has less delay than DM-MAC because of the static channel allocation. With DM-MAC, the delay is the highest, because the nodes in a single path need to switch channels more frequently. Figure 4(b) shows the channel utilization when the maximum fair throughput is achieved. TMCP achieves the minimum channel utilization, whereas it is at a maximum for LPMC. In case of TMCP, because of the static channel allocation, the channel utilization depends on the network topology. Therefore, the achieved throughput of different channels differs significantly. In contrast, DM-MAC and LPMC aim to use the smallest number of channels needed to satisfy the traffic load. As long as an acceptable delivery ratio is satisfied, LPMC does not add a new channel and achieves maximum channel utilization. Because of the added switching delay, DM-MAC cannot utilize the channel as fully as LPMC.

**Figure 5.**
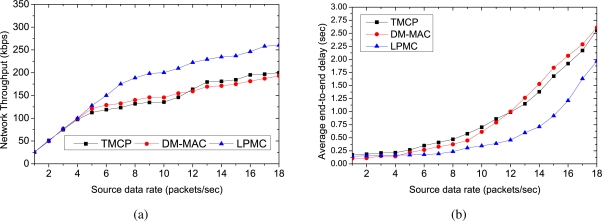
Performance comparisons for randomly selected 50 sources with different data rates when end-to-end reliability is considered (a) network throughput, (b) average end-to-end delay.

Figure 5 shows the results when end-to-end reliability is considered. In this case, the required reliability becomes 1 and lost packets are recovered by the end-to-end retransmissions. As shown in the Figure 5(a), with end-to-end reliability, the nodes using LPMC achieve a fair throughput up to a source rate of 7 packets/sec (where the network throughput is 176 kbps). Whereas, it is only 126 kbps for DM-MAC and 112 kbps for TMCP. Thereafter, all the mechanisms experience variations in achieved throughput because of the increased overhead caused by acknowledgments and end-to-end retransmissions. However, LPMC outperforms others as its load estimation policy (*i.e.*, the NLD module discussed in Section 3.3) can effectively estimate whether a channel is overloaded or not, even when end-to-end reliability is considered. DM-MAC’s load estimation only considers the local loss, and with the increased load of the end-to-end retransmission, most of the intermediate nodes are forced to change their channels (based on local condition), and they need to switch their channels for data forwarding. This increases the overhead, and decreases the network throughput. On the other hand, TMCP does not consider the load for its static channel allocation, and the channels become overloaded with the increased load of the end-to-end retransmissions. While LPMC can use the unused channels due to its dynamic channel allocation, TMPC’s static allocation does not allow the nodes to change their channels. Especially, when the nodes send their data at a high rate, or number of nodes from a smaller area send their data simultaneously, which might overload a particular channel and the nodes using that channel have a lower throughput. The dynamic channel allocation of LPMC can effectively divide the nodes into different channels and achieve a fair and efficient throughput, as long as the overall load is lower than the network capacity.

Figure 5(b) shows the average end-to-end delays incurred by the compared mechanisms as we increase the traffic load. We measure the average end-to-end delays of the successfully delivered packets. It is noticeable that average end-to-end delays of all the mechanisms with end-to-end reliability are higher than that of end-to-end delays (without considering end-to-end reliability as Shown in 4(a)). We measure that on an average only 7control overhead of the end-to-end retransmissions, overall network load increases which cause increased delay as compared to the absence of the end-to-end retransmissions. Initially, TMCP experiences higher end-to-end delays than that of DM-MAC and LPMC. Because of the static channel allocation of TMCP, the channels become overloaded fast and packets are lost due to increased congestion and contention, which increases the average end-to-end delays. On the other hand, when the traffic load becomes high, DM-MAC experiences more channel switching. This increased switching delay contributes to the increased end-to-end delay while using DM-MAC at higher traffic load.

**Figure 6.**
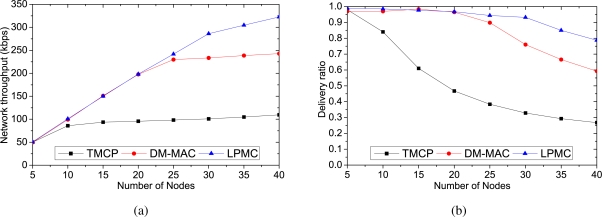
Performance with varying number of sources from a randomly selected location: (a) network throughput, (b) delivery ratio.

**Figure 7.**
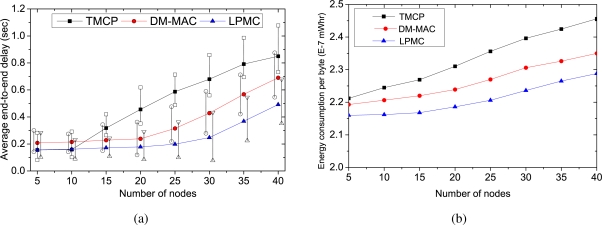
Performance with varying number of sources from a randomly selected location: (a) average end-to-end delay, (b) energy consumption per delivered data byte.

Figures 6 and 7 show the performance comparison for a varying number of sources from a randomly selected location. Each source generates data at a rate of 20 packets/s. We measure the impact of increasing the number of sources on various performance metrics. Due to the static channel allocation, some of the channels are kept idle in TMCP, achieving the minimum throughput with more than 10 sources, as shown in Figure 6(a). DM-MAC requires channel switching when multiple channels are active, and the network throughput is less than that of LPMC. Figure 6(b) shows the delivery ratio of the different mechanisms, and LPMC achieves the highest delivery ratio. In the case of TMCP, some channels are overloaded quickly, while others remain underutilized, because the sources are selected randomly. Therefore, packets tend to get lost, due to the increased congestion and contention, and the delivery ratio drops significantly as the number of nodes is increased. In contrast, both DM-MAC and LPMC add channels when the network becomes overloaded and, thus, achieve a higher packet delivery ratio.

Figure 7(a) shows the average end-to-end delays of the packets. Because TMCP does not utilize all of the channels, the network becomes overloaded when the number of nodes increases. This increases the packet loss rate and more packets tend to be retransmitted, which increases the average end-to-end delay. At higher traffic loads, DM-MAC adds new channels to share the traffic of an overloaded region. However, unlike LPMC, the nodes in DM-MAC need to frequently switch channels in a path. The channel switching increases the medium access time in DM-MAC, so the delay is higher than that of LPMC. Finally, Figure 7(b) shows the energy required for each mechanism to successfully deliver a data byte. Because of the increased packet delivery ratio, the energy consumption in LPMC is lower than that of DM-MAC and TMCP as we increase the number of nodes.

**Figure 8.**
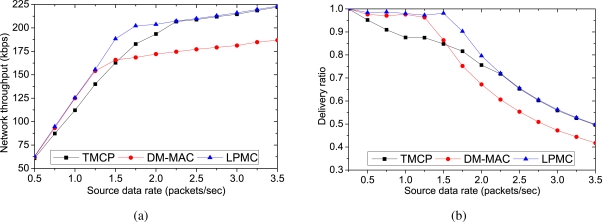
Impact of channel quality and external interference on the performance: (a) network throughput, (b) delivery ratio.

**Figure 9.**
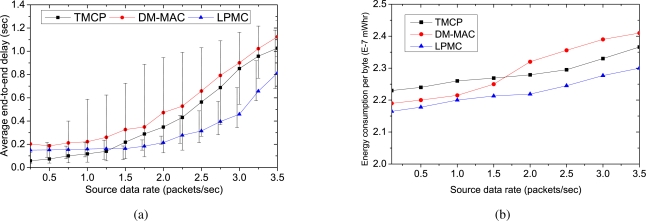
Impact of channel quality and external interference on the performance: (a) average end-to-end delay (The vertical lines shows the minimum and maximum delays for different channels), (b) energy consumption per delivered byte. All nodes send data to the sink.

Figures 8 and 9 demonstrate the impact of the channel quality and external interference. We vary the packet loss rate randomly from 10 to 80 percent. All of the nodes send their data to the sink. Both LPMC and DM-MAC assign a new channel when the channel quality degrades, whereas the nodes using the interfered (or low quality) channel cannot deliver their data in TMCP. More specifically, when the traffic load is low, LPMC and DM-MAC can find an unused or new channel to replace a low-quality channel. However, when the network becomes overloaded, these mechanisms cannot find an unused channel that can be added to the network. Therefore, LPMC and DM-MAC achieve the required delivery ratio, as shown in Figure 8(b) (and therefore, a higher throughput as shown in Figure 8(a)), as long as the offered load is less than or equal to the aggregate channel capacity. However, LPMC achieves higher throughput and reliability than DM-MAC, because it does not require channel switching. In contrast, the throughput in TMCP increases for higher source rates, because of the static channel assignment.

Figure 9(a) shows the average end-to-end delays when the offered loads are increased. As the traffic load is increased, all of the mechanisms tend to experience more delays. However, DM-MAC has the largest delay, due to the channel switching required by the nodes to forward or receive packets. Because all of the channels are active in TMCP, the delay is lower than that of LPMC for low data rates. However, as the traffic load increases, the nodes that are using low-quality channels experience a higher delay in TMCP. Furthermore, TMCP has a higher delay than LPMC for higher source rates, because the queuing and medium access delay is very high for low-quality channels. The vertical lines show the maximum and minimum average delays among the channels.

Finally, Figure 9(b) represents the energy consumption per byte as we increase the load of the network. Since the packet delivery ratio of LPMC is more tolerant of an increasing network load, it also proves to be more energy-efficient than DM-MAC and TMCP. As shown in the figure, energy consumption for each successfully delivered data byte in LPMC increases from 2.23 × 10^−7^ mWhr to only 2.36 × 10^−7^ mWhr as we increase the network load.

## 5. Conclusions

In this paper, we have designed a load-adaptive multi-channel communications system for WSNs. LPMC controls the channel allocation and deallocation at the sink and dynamically adds or removes channels. The dynamic channel allocation utilizes the limited channels of WSNs very efficiently and adds channel(s) only when necessary. LPMC increases the channel utilization and network throughput, and reduces the delay. LPMC works very efficiently in a dynamic environment in which traffic pattern changes very frequently, wireless environment is very dynamic, and even the presence of external interference might destroy the communications of one or more channels. LPMC dynamically divides a branch of the networks into smaller branches if the load of the branch is more than the capacity of a single channel. Finally, the simulation results demonstrate that LPMC outperforms the existing mechanisms.

In the future, we would like to test the performance of LPMC in a real testbed. Another future interest lies in designing a multi-channel communications scheme for large-scale WSNs where multiple sinks are considered.

## Figures and Tables

**Table 1.** System parameters used in simulation.

Parameter	Value	Parameter	Value

Link bit rate	250 Kbps	Packet size	32 Bytes
PHY Header	192 *μ*s	MAC Header	224 bits
ACK Packet	112 bits	Slot Time	20 *μ*s
SIFS	10 *μ*s	DIFS	30 *μ*s
Min CW	32	*R_req_*	0.95
No. of channels	6	Switching delay	200 *μ*s
*α*	0.12	*β*	0.10
